# Timelines of psychological, physical and sexual intimate partner violence among a nationally representative sample of Australian women

**DOI:** 10.1177/17455057251329640

**Published:** 2025-06-11

**Authors:** Elizabeth McLindon, Minerva Kyei-Nimakoh, Fiona C Giles, Kelly M FitzPatrick, Laura Tarzia, Kelsey Hegarty

**Affiliations:** 1 The University of Melbourne, Melbourne, VIC, Australia; 2 The Royal Women’s Hospital, Melbourne, VIC, Australia; 3 Murdoch Children’s Research Institute, The Royal Children’s Hospital, Parkville, VIC, Australia

**Keywords:** Coercive control, timelines of abuse, psychological violence, intimate partner violence

## Abstract

**Background:**

Violent and abusive behavior in relationships causes immense individual and community harm. Mapping the emergence of different types of abuse over time and recognising behavioral patterns, could enable more targeted intimate partner violence (IPV) prevention, screening, and early intervention. While there has been some qualitative research into early warning signs of abuse and escalating homicide risks in relationships, no known studies have asked a large sample of survivors to document the sequence of abusive behaviors in a recent relationship.

**Objectives:**

To explore timelines of psychologically, physically, and sexually abusive behaviors and life events in a recent relationship (during the last 5 years) to create a macro timeline of abuse.

**Design:**

Cross sectional survey of a nationally representative sample (gender, age, state, locality).

**Data sources and methods:**

Eight hundred and fifteen Australian IPV women survivors.

**Results:**

Results indicated that psychological abuse was a feature of most violent relationships, and almost always underpinned physical and sexual abuse. Psychological abuse often began before couples moved in together, while physical and sexual abuse came later. The earliest indicators for survivors that something was wrong was being isolated from others and feeling controlled by their partner. For many survivors, growing concern about the impact of abuse on their children occurred around the same time as leaving their relationship and trying to get help.

**Conclusion:**

Findings indicate a pattern of escalating behaviors over the course of a relationship that are consistent with the concept of coercive control. The macro timeline contains important learnings for intervening early with future survivors.

## Introduction

Intimate partner violence (IPV) or domestic abuse refers to relationships in which harmful psychological, sexual and physical behaviors are enacted to dominate and control a partner and create an environment of entrapment.^1,2^ How these abusive behaviors present in relationships over time has long been of interest to victim-survivors (hereafter referred to as “survivors”), clinicians, and researchers.^3–5^ Being able to map the order of behaviors and recognize patterns could enable more targeted IPV prevention, improved screening, and early intervention.^4^ The sequencing of psychological violence in the context of other IPV types and life events may further understandings of it as a possible “primary calculus of harm,” a theory posited by several researchers due to the prevalence of psychological IPV, its insidious and injurious impact for survivors, and its importance for determining lethality risk (p. 13).^5–8^

There has been some research into timelines and early warning signs of abuse in relationships. Several qualitative studies^3,5,6,9–14^ and one quantitative study,^4^ have explored the process of violence starting, how and when it accelerates, and how different types of violence interact. Within these studies, women have described their relationships as starting with usual romantic expectations,^5^ that moved into “love bombing” and sped-up commitment-seeking (i.e., moving in together, marriage, children) (p. 5).^3,5^ Excessive attention, possessiveness, jealousy, and isolation characterize this early relationship phase often labeled by perpetrators as displays of love,^6,9^ designed to disguise and excuse their belief in their right to control their partner.^3^ Dokkedahl et al.^7^ systematically reviewed research about psychological violence and its effects on mental health and described the continuum of psychological violence as beginning with aggressive behaviors such as yelling and insults, extending to coercive behaviors such as control and isolation. Charlot^4^ analyzed how frequently 147 participants experienced a long list of potential warning signs of abuse, including some behaviors by a partner that may not individually be considered abusive. The study employed machine learning to narrow more than 200 behaviors down to seven that were predictive of IPV.^4^ Warning signs were partners being arrogant or entitled, reacting negatively when they were denied something they wanted, disregarding the reasoning or logic of their partner because it disagreed with theirs, acting resentfully if questioned about their treatment of their partner, or creating an uncomfortable public situation, disagreeing about sexual matters, and pushing for sex even when their partner was not in the mood.^4^ A qualitative Canadian study with 30 survivors found that verbal abuse emerged early and was often normalized on the basis of community perceptions that blur fighting with abuse.^9^ While verbal violence sometimes cyclically escalated and de-escalated for months and years, the emergence of physical violence or the fear that abuse was increasing in severity, commonly prompted a turning point in how survivors saw their relationship.^9^ Similarly, phenomenological interviews with 15 survivors in a study by Queen, et al.^10^ found that behaviors engendering a sense of captivity (i.e., restricted access to friends, family, health care, or finances) appeared earlier than other behaviors. Survivors spoke of feeling fundamentally changed by these behaviours and dissociating from the reality to cope, describing the “defining” moment when they identified the behaviors as emotional abuse, usually after threats of harm, interventions by friends or family, or exposure to information about IPV.^10^ As survivors’ awareness of their partner’s manipulative tactics grew and they fought back or attempted to leave, the perpetrator’s violence increased in threat level and physical severity.^10^

Two studies in the United Kingdom have sought to understand escalating abuse and risks for intimate partner homicide using the “Domestic Homicide Timeline.”^3,5^ In a study of 372 homicide records, Monckton Smith^3^ found that domestic partner homicide was frequently preceded by a survivor withdrawing their commitment to the relationship, threatening the perpetrator’s dominance. In retaliation, the perpetrator escalated their use of coercive, threatening, and stalking behaviors, which ultimately lead to murder. In a smaller study with 12 survivors, Daw et al.^5^ found that once their relationship was established, survivors subtly noticed their partner’s character and behaviors changing in difficult to recognize or define ways, “you don’t notice it, it’s like boiling water” (p. 1).^5^ Verbal abuse, humiliation, and social restriction were followed by threats, intimidation, and punishments that would intensify and subdue until the onset of lethal violence or survivor suicide.^5^

To date, no known studies have used a large sample of survivors to document the sequential emergence of different abusive behaviors over time. Timelines are a way for survivors to visually organize narrative data in a chronological order, and sequence experiences in the context of social factors and major life events that were also present at the time.^14^ Timelines may have important stories to tell for intervening early with future survivors.^4^ While timelines have been used in some rich qualitative studies, an investigation of the academic literature (-2024) using the search and associate terms: “IPV”; “domestic violence”; “family violence”; “timeline*,” found no quantitative timeline studies with IPV survivors. The aim of this article was to address this gap in the evidence by answering the research question: “What is the order of psychologically, physically and sexually abusive behaviours by partners across a large nationally representative sample (gender, age, state, locality) of Australian survivors?”

## Method

This study was a descriptive, cross-sectional online survey conducted between 14 February and 5 April 2022. This article reports the subsample of participants who completed a timeline of abuse in their most recent abusive relationship.

### Participants

Participants were eligible for inclusion in this study if they were Australian English-speaking adult (aged 18 years and over) women survivors of recent IPV. Participants were registered panel members of an experienced commercial research company, iLink. iLink recruited a nationally representative sample on the demographics of gender, age, state, and locality (metropolitan or rural residence). A participation honorarium included panel points (paid by iLink in voucher form) and a draw to win one of two iPads.

### Full list of survey measures

Information accompanying the survey emphasised that it was voluntary, sensitive, and confidential. The full survey included measures of psychological, sexual, physical, and technology-facilitated IPV,^15–18^ nonpartner sexual assault and child abuse,^19^ current psychological and physical health,^20–24^ utilization of support and community services,^25^ and demographics.^19^ The survey took approximately 30 minutes to complete and included support information and optional guided self-care and well-being activities.

### Procedure

**Figure 1.**
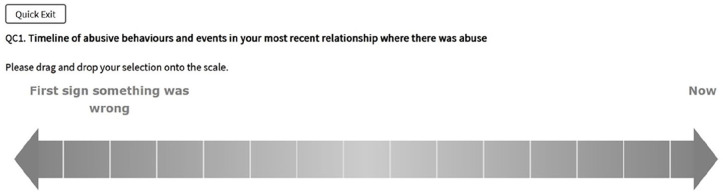
Blank timeline.

The study was predominantly quantitative with a small qualitative open-ended text component. The first study step was screening of potential participants for a recent history of IPV. Participants progressed to the full survey if they answered “yes” to one or more of seven behaviors by a partner or ex-partner sometime in the last 5 years: feeling afraid; having their day-to-day activities controlled; isolation from family or friends; monitoring, manipulative or harassing behaviours; threats to hurt them or others they cared about; being hit, slapped, kicked, or otherwise physically hurt; and, pressure or attempted pressure into unwanted sexual activity. Participants were encouraged to complete an optional electronic timeline of behaviors and events in their most recent abusive relationship and invited to take a personal photo of their completed timeline if this was felt to be of benefit. Specifically, participants were asked, “Looking at the list of behaviors and events underneath the timeline, could you drag and drop those that are relevant to you into a timepoint on the line. We would like you to think about the behaviors that were the first sign something was wrong in your relationship, as well as the behaviors and life events that appeared later. You will be able to add extra behaviors/events not already listed if you would like. It might be hard to remember all the things you think are important to go onto this timeline, which is normal and ok, just complete the timeline to the best of your memory.” The electronic timeline was a punctuated thick line with an arrow at both ends (Figure 1).

Underneath the blank timeline was a list of common psychological, sexual, and physical abusive behaviors (Table 1), and life events (Table 2). Participants were invited to select behaviors and events they had experienced and drag and drop them on their timeline in the order in which they first emerged. Three of the items were about children. The qualitative component of this study gave participants the choice to add up to three extra behaviors/events in their own words for placement anywhere on their timeline. Each point on the timeline was assigned a numeric code between 1 and 15. Behaviors/events that emerged around the same time could be placed at the same point in the timeline.

**Table 1.** List of abusive behaviors able to be placed on a timeline.

Type of abuse	Items
Psychological	*Controlled me* (e.g., Restricted access to food, phones, transport, healthcare, or controlled my day-to-day activities)
	*Isolated me* (e.g., Tried to keep me from socializing with family or friends or was obsessively jealous)
	*Verbally abused me* (e.g., Insulted me, put me down, or humiliated me in front of others)
	*Threatened me* (e.g., Threatened to hurt me, themselves, or others)
	*Monitored me* (e.g., Kept track of who I was with; monitored my time or stalked me)
	*Emotionally abused me* (e.g., Says I imagined things or denied their behavior; withheld affection)
	*Used my child/ren to manipulate me* (e.g., Tried to damage my relationship with my children, threatened to hurt them, or take them away from me)
Physical	*Hit me* (e.g., Hit or tried to hit me)
	*Hurt me* (e.g., Pushed grabbed or shoved me)
	*Used severe physical violence* (e.g., Beat me, strangled me, used knife, or gun)
Sexual	*Pressured me for sex* (e.g., Pressured me continually for sex even after I said “No” or demonstrated disinterest)
	*Made me have sex with them* (e.g., Used physical force to make me have sex or perform a sex act when I did not consent to it)

**Table 2.** List of life events able to be placed on the timeline.

Items
Left/separated or tried to (for any length of time)
Tried to get help or talked to someone about the abuse
Became concerned about the impact of abuse on my children
Realized that things would not change
Got married or moved in together
Gave birth

### Analysis

Data were analyzed using STATA 17.0 for Windows.^26^ Women with missing data for the primary outcome (timeline) were excluded from all analyses. Demographic characteristics for the sample were compared with Australian population data as a means of assessing the representativeness of the sample. Descriptive statistics of each abusive behavior and life event were examined to understand the distribution of the data (via the mean, standard deviation, median, and variance) and to assess departures from normality (via skew and kurtosis).^27^ Using the mean numeric position of each item, a “macro timeline” was developed (Figures 3 and 4). The phrase “macro timeline” refers to the sequence or order of abusive behaviors and events summarized using all participant timelines. The open-ended text of abusive behaviors that participants could add themselves was content-analyzed and the numeric position on the timeline examined. Open-text responses generally ranged in length from a short phrase to a sentence, necessitating a descriptive coding strategy to be implemented.^28–30^ Additional abusive behaviors/events added by participants were first checked to see whether they could be recoded under an existing behavior/event (i.e., where a participant was putting into their own words an abusive behavior/event that was in the default list). In the case of a participant adding to their timeline both a default behavior/event and an item in their own words that was later recoded by the researchers as a default behavior/event, the numeric position of the first instance was taken and additional positions omitted. Where an open-ended text addition did not overlap with an existing timeline behavior/event, an initial descriptive code was generated. Codes were inductively analyzed to understand concept connections and distinctions.

## Results

**Figure 2.**
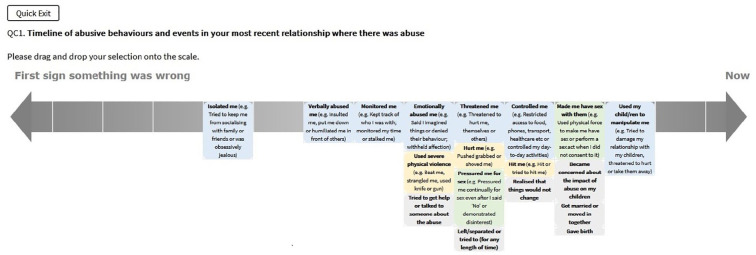
Example of completed timeline (participant ID 23491).

Of the 1026 survivor women participants who completed the full survey, 815 (79.4%) chose to complete a timeline of abusive behaviors and events during their most recent abusive relationship starting from the earliest signs that something was wrong (Figure 2).

The age of participants ranged from 18 to 83 years (*M* = 43 years). Most participants were tertiary educated (70.5%, 575), had a male partner (75.0%, 434/579) with whom they were currently living (61.0%, 497/815), and children (65.0%, 530/815). Participants predominantly lived in a metropolitan region of Australia (63.7%, 519/815) and were in paid employment (59.9%, 488/815) (Table 3). Around one in five participants (18.5%, 151/815) were born outside Australia, 9.1% (74/815) reported English was not their first language, and 5.6% (54/815) identified as Aboriginal and/or Torres Strait Islander. Around one in three (32.4%, 264/815) reported they were currently afraid of their partner or ex-partner, and the most common IPV type experienced in the last 12-months was severe combined physical, emotional, or sexual abuse (36.6%, 298/815).^17^

**Table 3.** Participant demographic characteristics (*N* = 815).

Demographic categories	All participants		Australian population (women)^a^
	*n*	%	%
Age^b^			
18–24 years	97	11.9	12.1
25–44 years	355	43.6	36.3
⩾45 years	363	44.5	51.6
Gender of partner^c^			
Male	434	75.0	59.8
Female	144	24.9	7.1
Nonbinary	1	0.2	0.2
Relationship status			
Living with a partner	497	61.0	68.0*
Not in a relationship	236	29.0	**
In a relationship, but not living together	82	10.1	**
Children			
Children living at home	299	56.4	48
Pregnant	28	3.4	**
State of residence^d^			
Victoria	248	30.4	25
New South Wales	219	26.9	32
Queensland	174	21.4	20
Western Australia	74	9.1	10
South Australia	69	8.5	7
Tasmania	15	1.8	2
Australian capital territory	13	1.6	2
Northern territory	3	0.4	1
Residential regionality^e^			
Metropolitan	519	63.7	66
Rural	268	32.9	34
Not specified	28	3.4	**
Indigenous status			
Aboriginal	45	5.5	3.9*
Torres Strait Islander	1	0.1	
Aboriginal and Torres Strait Islander	8	1.0	
Neither Aboriginal nor Torres Strait Islander	761	93.4	96.1
Birth country			
Australia	664	81.5	69.3*
Outside Australia	151	18.5	30.7
First language			
English	741	90.9	72.7*
Other	74	9.1	27.3
Highest education level			
Diploma or certificate	304	37.5	**
Degree or higher degree	271	33.3	36.0
Year 12 or lower	240	29.4	14.7
Employment status			
Fulltime (35+ h per week)	241	49.4	38.4
Part-time (<35 h per week)	247	50.6	44.0
Not currently employed	327	40.1	4.0
Low-income health care card	451	55.3	21.0

No missing data.

a Population data are weighted proportions from Australian Bureau of Statistics (ABS) (2021), other than age, state, and residential regionality which were provided by iLink. Values are percentages of women as a proportion of the total Australian population.

b ABS (2021) age population data provided by iLink (courtesy of Muriel Geagea) 30 September 2024.

c *n* = 579 participants reported being in a current relationship and responded to the partner gender question.

d ABS (2021) state of residence data provided by iLink (courtesy of Muriel Geagea) 30 September 2024.

e ABS (2021) residential regionality data provided by iLink (courtesy of Muriel Geagea) 30 September 2024.

* Gender-specific data not available.

** Comparable data not available.

### The macro-order of abusive behaviors

**Figure 3.**
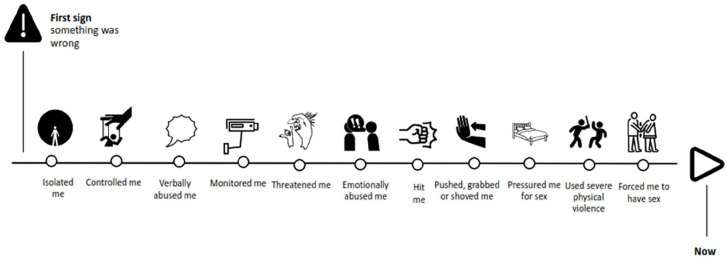
Macro timeline of abusive behaviors in most recent abusive relationship (*N* = 815) (ordered icons; not including child-related items).

The macro sequence of all abusive behaviors indicated that for the majority of the 815 participants, psychological abuse emerged before other types of IPV (see Table 4 for summary statistics and Figure 3 for visual macro timeline). Acts of psychological abuse were typically followed by physical, then sexual abuse.

**Table 4.** Macro timeline of abusive behaviors and events—summary statistics^a^.

Behavior/event	All participants (*N* = 815)	Participants with children (*n* = 530)	Participants without children (*n* = 285)
Isolated me			
Mean (SD)	4.65 (3.0)	4.70 (3.0)	4.57 (2.9)
Median	4.0	4.0	4.0
*n* (%)	473 (58.0)	311 (58.7)	162 (56.8)
Controlled me			
Mean (SD)	4.87 (3.3)	5.00 (3.3)	4.66 (3.2)
Median	4.0	4.0	4.0
*n* (%)	411 (50.4)	262 (49.4)	149 (52.3)
Verbally abused me			
Mean (SD)	5.36 (3.3)	5.47 (3.5)	5.15 (2.9)
Median	5.0	5.0	5.0
*n* (%)	547 (67.1)	359 (67.7)	188 (66.0)
Monitored me			
Mean (SD)	5.85 (3.4)	6.09 (3.4)	5.43 (3.4)
Median	5.0	6.0	5.0
*n* (%)	425 (52.1)	266 (50.2)	159 (55.8)
Got married or moved in together			
Mean (SD)	5.86 (4.1)	5.51 (3.0)	6.82 (4.2)
Median	5.0	5.0	7.0
*n* (%)	265 (32.5)	193 (36.4)	72 (25.3)
Threatened me			
Mean (SD)	5.88 (3.0)	5.87 (3.1)	5.91 (3.0)
Median	6.0	6.0	5.5
*n* (%)	424 (52.0)	274 (51.7)	150 (52.6)
Emotionally abused me (gaslit, withheld affection)			
Mean (SD)	5.89 (3.5)	6.14 (3.6)	5.45 (3.3)
Median	5.0	5.0	5.0
*n* (%)	602 (73.9)	385 (72.6)	217 (76.1)
Hit me			
Mean (SD)	6.03 (3.4)	5.90 (3.2)	6.29 (3.7)
Median	6.0	6.0	6.0
*n* (%)	310 (38.0)	207 (39.0)	103 (36.1)
Pushed, grabbed or shoved me			
Mean (SD)	6.30 (3.2)	6.27 (3.3)	6.37 (3.1)
Median	6.0	6.0	6.0
*n* (%)	352 (43.2)	229 (43.2)	123 (43.2)
Pressured me for sex			
Mean (SD)	6.88 (3.3)	7.11 (3.4)	6.55 (3.1)
Median	7.0	7.0	7.0
*n* (%)	378 (46.4)	224 (42.3)	154 (54.0)
Used severe physical violence (Beat, strangled, used weapon)			
Mean (SD)	7.00 (3.5)	6.94 (3.7)	7.11 (3.3)
Median	7.0	7.0	7.0
*n* (%)	238 (29.2)	154 (29.0)	84 (29.5)
Gave birth			
Mean (SD)	*	7.05 (3.8)	*
Median	*	7.0	*
*n* (%)	*	168 (31.7)^b^	*
Used my child/ren to manipulate me			
Mean (SD)	*	7.19 (3.5)	*
Median	*	7.0	*
*n* (%)	*	238 (44.9)	*
Made me have sex with them (used force)			
Mean (SD)	7.51 (3.3)	7.28 (3.4)	7.86 (3.1)
Median	8.0	8.0	8.0
*n* (%)	281 (34.5)	173 (32.6)	108 (37.9)
Became concerned about the impact of abuse on my children			
Mean (SD)	*	8.38 (3.3)	*
Median	*	8.0	*
*n* (%)	*	173 (32.6)	*
Left/separated or tried to			
Mean (SD)	8.38 (3.4)	8.17 (3.5)	8.72 (3.4)
Median	8.0	8.0	9.0
*n* (%)	376 (46.1)	232 (43.8)	144 (50.5)
Tried to get help or talked to someone about the abuse			
Mean (SD)	8.48 (3.3)	8.42 (3.2)	8.59 (3.6)
Median	9.0	8.0	9.0
*n* (%)	284 (34.8)	189 (35.7)	95 (33.3)
Realized that things would not change			
Mean (SD)	9.28 (3.3)	9.37 (3.3)	9.10 (3.2)
Median	9.5	10.0	9.0
*n* (%)	398 (48.8)	264 (49.8)	134 (47.0)

SD: standard deviation; *n* (%): number and proportion of participants who placed item on their timeline.

a Based on timeline with 15 timepoints.

b While 530 participants who completed a timeline had children, only 168 dragged and dropped “gave birth” onto their timeline, indicating that around one in three had a baby during the relationship upon which their timeline was based (i.e., their most recent abusive relationship).

* Child-related item.

**Figure 4.**
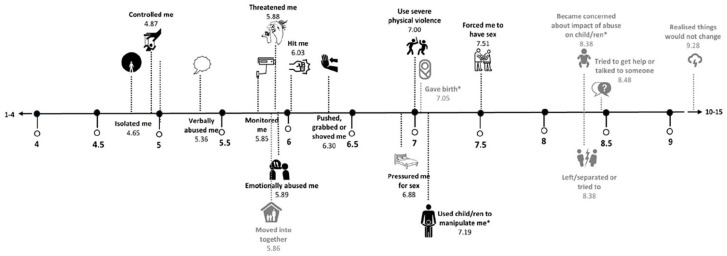
Macro timeline of abusive behaviors and events in most recent abusive relationship (*N* = 815). *Child-related item (*n* = 530).

When placing life events in the context of abusive behaviors, the macro timeline indicated that psychological abuse often began before couples got married or moved in together, while physical and sexual abuse came afterward (Figure 4). For many survivors, growing concern about the impact of the abuse on their children occurred around the same time as leaving their relationship, trying to get help, before realizing that things would not change.

### Differences between the timelines of survivors with and without children

Participants with children, on average, reported that the perpetrator used their child/ren to manipulate them (*M =* 7.19, Median = 7.0) comparatively soon after giving birth (*M =* 7.05, Median = 7.0). The principal difference between the timelines of participants with and without children was the macro order (between 1 and 15) in which behaviors/events occurred. Participants with children, who tended to be older than participants without children, indicated that they had gotten married or moved in with their partner earlier in the sequence of abusive behaviors (*M =* 5.51, Median = 5.0) than participants without children (*M* = 6.82, Median = 7.0). Participants with children noticed that on average, physical, and sexual violence emerged after they had moved in with their partner, while participants without children typically got married or moved in together after psychological, physical, and sexual violence abuse had already emerged. Further, participants with children appeared to leave/separate from their partner at an earlier timepoint than participants without children (*M* = 8.17, Median = 8.0 compared to *M* = 8.72, Median = 9.0).

### Additional timeline behaviors and events

Of 815 participants who completed a timeline, 171 added an additional abusive behavior or event in their own words. Participants could add up to three additional behaviors/events and 170 additions were made in the first optional space, 78 in the second, and 44 in the third. A total of 232 timeline additions were made, however, during analysis most of these added behaviors/events (157/232) could be recoded under an existing behavior/event. For example, one participant added the behavior: “*Reduced my access to money*” (ID: 19774), while another wrote, *“Not allowing use of car”* (ID: 6475) and both were recorded a, C*ontrolled me*. Content analysis resulted in the generation of three additional timeline behaviors that did not overlap with any of those provided. These were: *Perpetrator cheated on me, Perpetrator used alcohol and/or other drugs*, and *Technology-facilitated abuse* (Table 5). A further 47 open-ended text behaviors/events added by participants were so disparate that they did not form a theme (i.e., “*He was so in love with me and didn’t want to lose me*” [ID: 814]; “*My paid work was ignored*” [ID: 8055], “*Was not happy at all that none of our children were boys*” [ID: 9708] etc). Since most of the additional behaviors/events added by participants either overlapped with existing behaviors/events or did not comprise a distinct theme, while the three additional behaviors were suggested by fewer than 14 people, no additions were made to the macro timeline. Analysis suggested that many participants used the additional behaviors/events function to share nuance, detail, or contextual information unique to their experience, rather than suggesting a new behavior/event.

**Table 5.** Three timeline additions.

Theme name	Number of participants within theme	Mean position of theme on timeline (1–15)	Open-ended text examples of theme
Perpetrator cheated on me	13	11.77	“Cheated on me” (ID: 8982)
			“When he started using dating apps while still in the relationship” (ID: 5573)
			“Unfaithful” (ID: 3177)
Perpetrator used alcohol and/or other drugs	12	11.33	“Alcohol-induced behavior” (ID: 34480)
			“Extreme alcohol abuse” (ID: 26196)
			“Refused to stop bringing drugs into my house when I was trying to stop using and had asked for help. . .” (ID: 3885)
Technology-facilitated abuse	3	7.66	“Phone harassment” (ID: 8798)
			“Multiple messages and calls a day” (ID: 9414)
			“Sending naked pictures of me” (ID: 38507)

### Early warning signs

For all participants, irrespective of whether they had children, the earliest indicators that something was wrong was being isolated from others (*M* = 4.65, Median = 4.0) and feeling controlled by their partner (*M* = 4.87, Median = 4.0).

### Psychological abuse most common

Indicating how pervasive experiences of psychological abuse may be within IPV relationships, 91.5% (746/815) of participants dragged one or more psychologically abusive behaviours onto their timeline, while around half-placed act/s of physical (49.3%, 402/815) or sexual (48.8%, 398/815) IPV. Where a participant had experienced physical and/or sexual abuse, psychological abuse had nearly always already been present: only 3.2% (26/815) of participants placed physical or sexual items on their timeline without psychological items. Nearly a third of participants (30.8%, 251/815) dragged and dropped only psychologically abusive behaviors onto their timeline. The most frequently cited abusive behaviors were emotional abuse (gaslighting or withholding affection) placed on the timelines of 73.9% (602/815) of participants, verbal abuse (67.1%, 547/815), and isolation from others (58.0%, 473/815).

## Discussion

This is the first known nationally representative (gender, age, state, locality) and quantitative study in which women retrospectively constructed their own timelines of a recent abusive relationship. Analysis resulted in a macro timeline presenting a mean score timepoint of the abusive behaviors and events experienced by all participants. This study starkly demonstrates the concept of coercive control as a sequence of escalating behaviors over time.^6^

The macro timeline indicated that the early warning signs of abuse are: isolation, controlling behaviors, and verbal abuse. These were followed by a constellation of monitoring acts, threats, and emotional abuse. Emotional abuse (gaslighting, being frozen out) and verbal abuse (insults, humiliation), were tactics that participants most frequently dragged onto their timelines. Characteristics of abusive relationships consistent with the timeline findings have to some extent been qualitatively described before.^3,5,6,9–14^ The perpetration of psychological entrapment generally occurred before couples got married or moved in together, perhaps during the accelerated commitment-seeking phase described in previous literature.^3,5^ For the survivors with children in this study, physical and sexual abuse often first emerged after they had made a commitment to their partner either through marriage or cohabitation. The macro timeline indicated that, on average, childbirth was followed by increasingly severe sexual violence. This is consistent with other studies that have found changes or escalations to women’s experience of violence in the years after childbirth.^8,12,31^ For many survivors, concern about the impact of the abuse on their children occurred around the same time as they left their relationship and tried to get help, which was preceded by identifying that their child/ren were being used to manipulate them. Help seeking appeared toward the end of the macro timeline, after trying to leave the relationship, which may signify the multiple barriers to support that are faced by survivors, as has been canvassed in the literature, including fear, shame, lack of information, and concern about not being believed.^25^

The macro timeline enables a quantitative visual sequence and progression in levels of coercive control over the survivor, beginning with attacks to her mind, then her physical body, then her sexual self, her core.^32^ The first set of tactics were psychological, breaking connection with others, freedom, safety, reality, and sense of self. Nonphysical violence was perpetrated against nearly all survivor participants, reinforcing the concept that psychological tactics may be considered a foundational harm.^5,6^ Consistent with previous research, for about half of the survivor participants in this study, psychological coercion appeared to provide the enabling environment in which physical and/or sexual violence could be introduced and maintained.^6,7,32^ Research with survivors has highlighted the loss of agency, autonomy, self-trust, and self-belief that psychological tactics engender, leading to mental entrapment.^2,33^ The second set of behaviors used against survivors in the macro timeline encompassed attacks to their body. The onset of physical violence—hitting, pushing, grabbing, and shoving—may signal an escalation in coercive control and perpetrator dominance as the survivor is both literally constrained and mentally diminished through the bodily violence.^9^ The final tactic to emerge in the macro timeline was the attempt to control the survivor’s spirit via sexual violence. Survivors of intimate partner sexual violence have described it in previous research as a deeply dehumanizing and uniquely harmful form of abuse that “kills something inside you” and causes “damage from the inside out” (pp. 1 and 10).^34^ The macro sequence of abusive behaviors suggests that the stepped introduction of coercive violations beginning with separating the survivor from others and becoming increasingly intrusive and degrading to the point of sexual violence.

### Study strengths and limitation

A strength of this study was that its nationally representative sample (gender, age, state, locality), size and quantitative method enabled a macro timeline to be developed. However, the sample may differ from the broader population in other important ways (i.e., comparatively lower educational attainment and higher proportion of low-income health care card holders). The macro timeline represents only the mean order of abusive behaviors and events; individual timelines among participants did differ in many and meaningful ways. The timeline format was developed to address the identified gap in current knowledge regarding the sequence in which abusive behaviors often emerge. Timelines could not, however, illuminate how long into the relationship (days/months/years) behaviors first emerged, the duration for which they were present, their dormancy, and recurrence or oscillating intensity, nor the broader dynamics of each relationship. Further, the list of behaviors/events provided was neither exhaustive nor nuanced, particularly across the physical and sexual abuse items.^4,14^ The timeline does not illuminate how often leaving/returning may have occurred, although literature indicates this to be a cycle common to many abusive relationships.^3^ While timelines had the capability for participants to add up to three additional items, it is possible that some important warning signs of abuse were missed from this method. However, most of the timeline additions supplied by participants overlapped in meaning with the provided list of behaviors/events, suggesting they were adequate.

### Implications

This research evokes clear opportunities for prevention and early intervention, including better community education about psychologically abusive behaviors which occur early in relationships, particularly aimed at young women. Training for health and helping professionals to look for signs and ask about psychological abuse when people are contemplating life transitions, may also be effective. These findings underscore the importance of asking about psychological violence during routine prenatal care in primary and tertiary healthcare and sharing information about how children may be impacted by violence and abuse. Future research should take an intersectional lens to understanding patterns of abuse among different groups of survivors. Greater functionality of the timeline format that could address some of this study’s limitations would also be useful.

## Conclusion

Patterns of psychological, physical, and sexual abuse can, over time, create social entrapment, a fearful environment, mental anguish, and a diminishing sense of self-worth for survivors. The identification of psychological IPV as the earliest sign of potentially escalating coercive control, is urgent, particularly for women prior to embarking upon life transitions such as moving in with their partner or having a baby. Beyond the individual, the systems and services with whom survivors and people who use violence interact, need to better identify and respond to psychological abuse, given the deleterious bio-psychosocial harms of IPV throughout the life course.

## Declarations

**Ethics approval and consent to participate:** The research conducted in accordance with the Declaration of Helsinki and was approved by the University of Melbourne Human Research and Ethics Committee (ID: 2680). Informed consent was obtained from all recipients prior to the study.

**Consent for publication:** Not applicable.

**Availability of data and materials:** Due to confidentiality issues, the data are not publicly available but can be obtained from the authors upon reasonable request, contact author EM.
